# A synergistic framework integrating global context and structural features for breast ultrasound lesion detection

**DOI:** 10.3389/fonc.2026.1857024

**Published:** 2026-06-26

**Authors:** Xiangqiong Wu, Yujie Tang, Yaxuan Zhou, Peng Wang

**Affiliations:** 1 School of Computer Science, Hunan First Normal University, Changsha, China; 2 School of Computer Science and Technology, Xidian University, Xi’an, China; 3 School of Electronic Information, Hunan First Normal University, Changsha, China

**Keywords:** attention mechanism, breast ultrasound, lesion detection, Mamba, medical image detection

## Abstract

**Background:**

Accurate breast lesion detection in ultrasound images remains challenging due to speckle noise, acoustic artifacts, low contrast, and blurred lesion boundaries. Although YOLO-based detectors are efficient, they may not fully capture long-range contextual dependencies and directional structural information that are important for reliable lesion localization.

**Methods:**

We proposed a lightweight context-structure synergistic framework based on YOLOv13. A Dual-Stream Mamba Aggregation (DSMA) module is introduced to enhance contextual feature aggregation with linear-complexity state-space modeling, while a Structure-aware Axial Attention (SAA) module is used to model horizontal and vertical structural dependencies. The two modules are integrated in a stage-specific manner to improve feature representation with limited computational overhead.

**Results:**

On the BUV and WH-BUS datasets, the proposed method achieved competitive detection performance while maintaining 2.50M parameters, 6.4 GFLOPs, and 161.29 FPS. Ablation, cross-dataset, robustness, and visualization analysis showed that DSMA and SAA provide complementary benefits for contextual representation and structure-aware localization.

**Conclusion:**

The proposed method provides a lightweight detection framework for breast ultrasound images by jointly modeling contextual and structural features.

## 1 Introduction

Breast cancer remains one of the most prevalent malignancies among women worldwide International Agency for Research on Cancer (1), and early detection is essential for timely intervention and improved prognosis. Ultrasound is widely adopted in clinical practice for its safety, accessibility, and superior performance in evaluating dense breast tissue. However, accurate lesion detection in ultrasound images remains challenging due to speckle noise, acoustic shadowing, low contrast, blurred boundaries, and heterogeneous tissue appearances. In routine clinical practice, lesion localization and interpretation still depend heavily on radiologist experience, which may lead to inter-observer variability. Therefore, developing an efficient and reliable computer-aided detection method for breast ultrasound images has practical significance for improving diagnostic consistency and workflow efficiency.

With the rapid advancement of deep learning, convolutional neural networks (CNNs) have been widely applied to image detection and have achieved promising performance Sun et al. (2)Li et al. (3) Xiao et al. (4) Wang and Yao (5) Jiang et al. (6). Existing object detection methods can be broadly categorized into two-stage and one-stage paradigms. Two-stage detectors, such as the R-CNN family Ren et al. (7) Liu et al. (8), rely on region proposal generation followed by classification and localization refinement. Although these methods can achieve high detection accuracy, their relatively high computational cost and latency limit their practicality in real-time clinical deployment. In contrast, one-stage detectors, including SSD Liu et al. (9), FCOS Tian et al. (10), and YOLO series Wu et al. (11) Tian et al. (12) Lei et al. (13), directly predict object locations and categories, offering a better balance between accuracy and efficiency.

Therefore, YOLO-based models Tian et al. (12)Lei et al. (13)Varghese and M. (14) Wang et al. (15) Khanam and Hussain (16) have become widely used for real-time detection. Recent efforts have focused on enhancing feature representation for ultrasound-specific challenges. For instance, Wang et al. Wang et al. (17) incorporated oriented bounding boxes and deformable convolutions based on YOLOv8s to capture irregular lesion shapes and boundary information. Li et al. Li et al. (18) introduced a multi-scale module and a detail-enhanced detection head into YOLOv8 to address the real-time detection demands of embedded terminal devices for breast ultrasound. Gui et al. Gui et al. (19) developed the FSYOLOv9 model customized for breast cancer detection, which incorporated an extra max-pooling layer and a high-frequency Haar wavelet convolution kernel to enhance morphology and texture representation. Wu et al. (20) embedded anatomical prior knowledge through programmable gradient information and large-kernel convolution to expand the receptive field. Despite these improvements, CNN-based detectors are still mainly constrained by local receptive fields, making it difficult to distinguish lesion-related structures from artifacts or background textures when global contextual information is insufficient.

To address the limitation of local convolution, global context modeling has attracted increasing attention. Transformer-based detectors introduced self-attention mechanisms to model long-range dependencies and have shown strong performance in various vision tasks. Representative models, such as RT-DETR Zhao et al. (21), provide an end-to-end object detector with improved efficiency compared with conventional DETR-style methods. Chen et al. Chen et al. (22) introduced a simple yet efficient DETR training approach that introduces a group-wise way for one-to-many assignment. CASCADE Rahman and Marculescu (23) exploited hierarchical transformer-based features and adopted multi-stage feature and loss aggregation to improve detection performance. In medical imaging, hybrid CNN-Transformer models have also been explored to combine local feature extraction with global dependency modeling. For example, Chen et al. Chen and Lu (24) proposed a hybrid CNN-Transformer framework that combines local feature extraction with long-range dependency modeling, enhancing the detection of complex microlesions. Tao et al. Tao et al. (25) introduced a DETR-based model for 3D ABUS lesion detection, combining the multi-view co-attention mechanism with unsupervised contrastive learning. Gu et al. Gu et al. (26) proposed a saliency-guided selection-driven Multi-Scale DEtection TRansformer network, which adaptively captures breast lesion features with varying sizes and spatial distributions by multi-scale dynamic convolution. However, standard self-attention introduces considerable computational cost for high-resolution feature maps. In addition, global attention aggregates information from all spatial positions, which may introduce irrelevant responses from speckle noise, acoustic artifacts, or background tissues in low-contrast ultrasound images. Recently, state-space models (SSMs) have emerged as an efficient alternative for long-range dependency modeling with linear complexity Wang et al. (27). SSM-based visual models Chen et al. (28)Wei et al. (29)Huang et al. (30) can provide a global receptive field while maintaining computational efficiency, making them potentially suitable for medical image analysis involving large feature maps. Nevertheless, directly applying global sequence modeling to breast ultrasound detection may still be insufficient, because lesion localization depends not only on semantic context but also on fine-grained structural continuity and boundary-related information.

In parallel, attention mechanisms have been widely used to enhance feature representation. For example, SENet Hu et al. (31) introduced channel-wise attention by modeling inter-channel dependencies, while CBAM Woo et al. (32) further incorporated spatial attention with minimal overhead. ECA Wang et al. (33) adaptively selected the kernel size of 1D convolution to achieve local cross-channel interaction, and CA Hou et al. (34) embedded positional information into channel attention. Song et al. Song et al. (35) presented a global-local attention module that incorporates all four forms of attention: local and global, spatial and channel. SKNet Li et al. (36) dynamically adjusted receptive fields through selective kernel fusion, and LSKNet Li et al. (37) extended this concept by integrating large-kernel convolutions with spatial attention. For medical imaging tasks, ESKNet Chen et al. (38) proposed an enhanced selective kernel convolution for breast lesion segmentation, enabling adaptive recalibration across channel and spatial dimensions. These approaches improve feature representation to some extent, but many of them rely on global weighting or isotropic aggregation. This design may be less effective for breast ultrasound images, where lesion morphology, boundary continuity, posterior acoustic patterns, and tissue interfaces often exhibit directional characteristics.

Therefore, breast ultrasound lesion detection requires a framework that can simultaneously model contextual dependencies and preserve structural information. Excessive semantic aggregation may suppress subtle lesion boundaries, whereas insufficient contextual modeling may make the detector sensitive to artifacts and heterogeneous background textures. To address this issue, we propose a lightweight context-structure synergistic detection framework based on YOLOv13. The proposed Dual-Stream Mamba Aggregation (DSMA) module enhances long-range contextual representation through efficient state-space feature aggregation, while the Structure-aware Axial Attention (SAA) module models directional structural dependencies along horizontal and vertical axes. By combining these two complementary modules, the proposed framework aims to improve lesion localization under noisy and low-contrast ultrasound conditions while maintaining real-time efficiency.

The main contributions of this work are summarized as follows:

- We propose a lightweight context-structure synergistic framework for breast ultrasound lesion detection, which jointly models long-range contextual representation and directional structural information under noisy and low-contrast conditions.
- We design a DSMA module to enhance contextual feature aggregation with linear-complexity state-space modeling, and an SAA module to improve structure-aware representation through axial attention along horizontal and vertical directions.
- Experiments on two public breast ultrasound datasets, including ablation studies, cross-dataset evaluation, robustness analysis, and feature visualization, showed that the proposed framework achieved competitive detection performance with limited computational overhead.

## 2 Materials and methods

### 2.1 Datasets and evaluation metrics

To develop and rigorously evaluate the proposed method, we utilized two publicly available breast ultrasound datasets collected from different clinical centers, enabling assessment of cross-center generalization. The first is the BUV dataset Lin et al. (39), introduced in Lin et al. (39). This dataset consists of a total of 24629 images from a single hospital. All images are acquired by LOGIQ-E9 and PHILIPS TIS L9-3. Two pathologists with eight years of experience in breast pathology were invited to manually annotate the breast lesion rectangles inside of each image. Following the official partition, we used 20188 images for training and 4441 images for testing. The second is the WH-BUS (Wuhan Breast Ultrasound) dataset Zhang et al. (40), collected by Zhang et al. (40), which contains 20430 images. In our main experiments, we employed the entire WH-BUS dataset as an independent external test set to evaluate zero-shot cross-center generalization performance, thereby ensuring strict separation from the training data. To further investigate the generalization under distribution shifts, we conducted bidirectional cross-dataset experiments. We split the WH-BUS dataset into a training subset of 13665 images and a test subset of 6765 images. Models were trained on one dataset and tested on the other without any fine-tuning. Although both datasets are public and retrospective, they were acquired at different hospitals with variations in ultrasound equipment, scanning protocols, and patient demographics. This setup provides a reasonable proxy for multi-center validation. However, we acknowledge that prospective evaluation on additional clinical data from more diverse centers would further strengthen the clinical translation potential of the proposed method.

To comprehensively evaluate the computational efficiency and detection performance of the proposed framework, we quantified the model complexity using the number of parameters (Params) and floating-point operations (FLOPs) while measuring inference speed in frames per second (FPS). These metrics ensure that the framework remains lightweight and suitable for real-time clinical deployment in breast ultrasound analysis. For detection performance, we adopted Precision and Recall as fundamental metrics. In the context of breast ultrasound imaging, Precision quantifies the model’s ability to suppress false positives, whereas Recall measures sensitivity in detecting true lesions, which is particularly critical for minimizing missed diagnoses in early breast cancer screening.

Furthermore, to further assess localization accuracy under the challenging conditions of ultrasound images, we utilized mean average precision (mAP) at two intersection-over-union settings. Specifically, mAP50 evaluates the model’s overall capability to detect the presence of lesions with a moderate localization requirement. More stringently, we computed mAP by averaging the mean average precision across intersection-over-union thresholds from 0.50 to 0.95 in steps of 0.05. This stricter metric imposes progressively higher demands on spatial overlap, providing a robust evaluation of the model’s precision in localizing lesions with ambiguous and irregular boundaries typical of breast ultrasound images.

### 2.2 Implementation details

All experiments were implemented based on the Ultralytics YOLO framework using PyTorch with Python 3.11 and CUDA 11.8. Input ultrasound images were uniformly resized to 640×640 pixels. The models were trained using the SGD optimizer with an initial learning rate of 0.01 and a batch size of 8. The momentum and weight decay were set to 0.937 and 0.0005, respectively. A warm-up strategy was adopted during the first 3 epochs, where the learning rate was linearly increased from 0 to 0.01, and the warm-up momentum was initialized to 0.8. The default YOLO detection losses were employed, including box regression loss, classification loss, and distribution focal loss, with corresponding loss weights of 7.5, 0.5, and 1.5, respectively. To ensure fair comparison across methods, all models were trained from scratch without using external pre-trained weights such as ImageNet or COCO. This setting was adopted mainly because breast ultrasound images differ substantially from natural images in both appearance and statistical characteristics. Using pre-trained weights from natural-image datasets may therefore introduce additional biases that are difficult to quantify. In addition, training all models under the same initialization setting allows the observed performance differences to be attributed more directly to the proposed architectural modifications, rather than to the benefits of pre-training. Considering the scale of the training datasets used in this study, training from scratch was sufficient for stable convergence and enabled the models to adapt specifically to ultrasound imaging characteristics, including speckle noise and lesion appearance patterns.

For data augmentation, HSV augmentation was applied with hue, saturation, and value adjustment ranges of (± 0.015), (± 0.7), and (± 0.4), respectively. Additional augmentation strategies included random translation (± 0.1), random scaling (± 0.5), horizontal flipping with a probability of 0.5, mosaic augmentation with a probability of 1.0, and copy-paste augmentation with a probability of 0.1 using the flip mode. RandAugment was adopted as the automatic augmentation policy, and random erasing was applied with a probability of 0.4. Following the default Ultralytics training strategy, mosaic augmentation was disabled during the final 10 epochs to improve training stability and convergence consistency.

To improve experimental reproducibility and evaluate optimization stability, the main quantitative comparison experiments were independently repeated five times using different random seeds (42, 666, 1024, 2025, and 3407), and the corresponding results were reported as mean ± standard deviation. For the remaining experiments, including robustness analysis, module position and replacement, and visualization analysis, a fixed random seed (seed=0) was adopted to ensure consistent experimental settings and fair comparisons across different module configurations.

In addition, to prevent model overfitting and improve training efficiency, an early stopping strategy was employed in this study. The maximum number of training epochs was set to 500, and model validation was performed after each epoch. Training was automatically terminated if the validation loss did not decrease for 100 consecutive epochs. Consequently, the actual training process typically converged between 114 and 164 epochs. The estimated training time for a complete run ranged from approximately 10 to 31 hours using a single NVIDIA RTX 4090 GPU.

### 2.3 Network architecture

**Figure 1:**
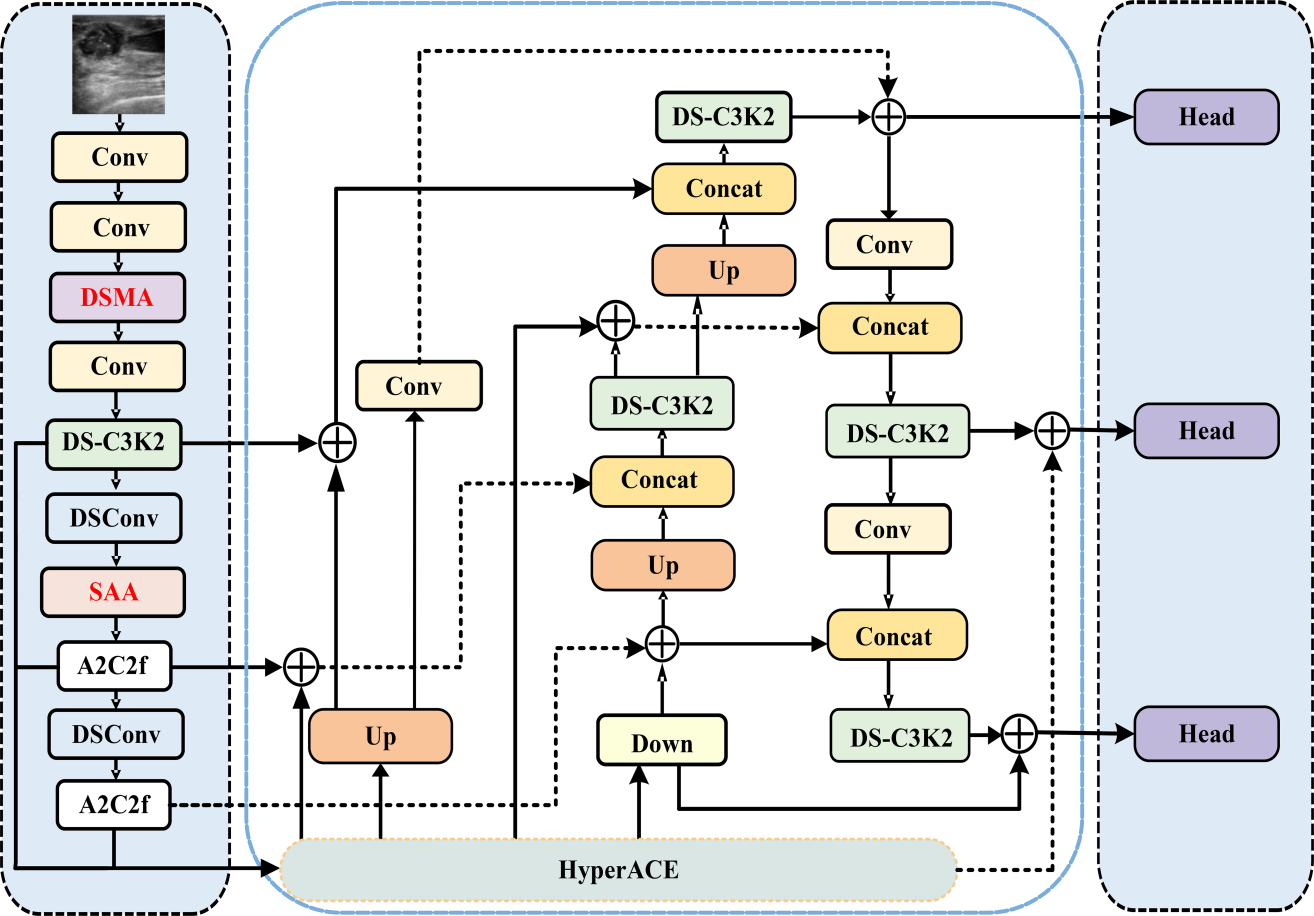
Overall architecture of the proposed breast ultrasound lesion detection framework.

**Figure 1** illustrates the overall architecture of our proposed object detection framework. Given a breast ultrasound image, features are hierarchically extracted through the backbone network. To achieve accurate lesion localization while mitigating the interference of speckle noise, the DSMA and SAA modules are strategically integrated into the shallow and deep stages of the network, respectively.

Specifically, the DSMA module, designed based on the DSC3K2 structure, is deployed in the early, shallow feature extraction stages. Since shallow layers possess high-resolution feature maps but lack a global receptive field, the DSMA module is introduced to capture long-range contextual dependencies at early feature extraction stages with linear computational complexity, while preserving rich local details via its progressive aggregation mechanism. In contrast, the SAA module is integrated into the subsequent deeper layers. In deep stages, spatial resolution is substantially reduced, making lesion boundaries ambiguous for accurate bounding box regression. The SAA module explicitly models directional spatial dependencies along orthogonal orientations, which helps strengthen structural features and reduce responses from non-lesion background patterns. Finally, the refined multi-scale features are fed into the detection head to predict the bounding boxes and category probabilities of the lesions.

### 2.4 Dual-stream Mamba aggregation

**Figure 2:**
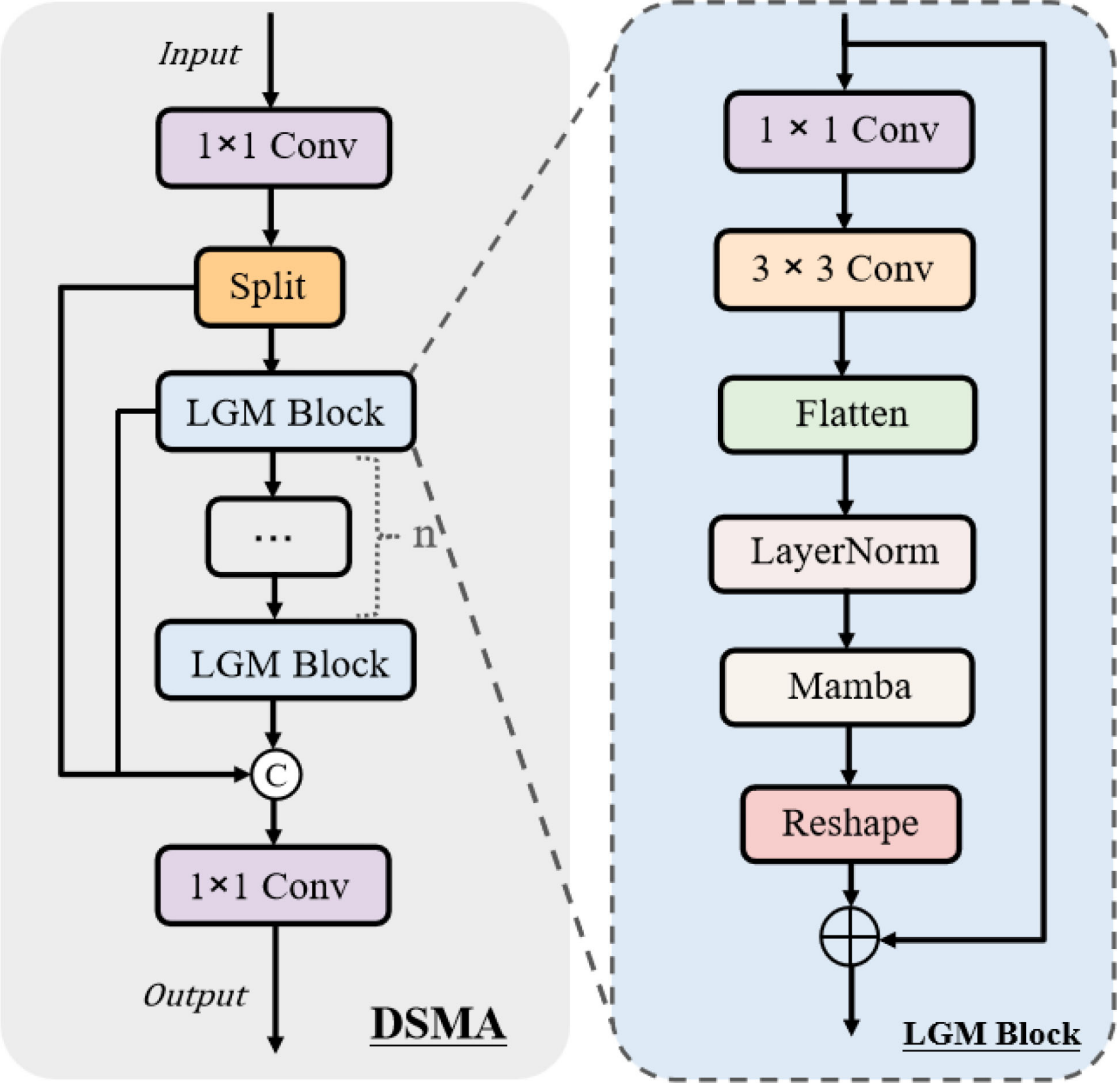
Overall architecture of the proposed dual-stream Mamba aggregation module.

As shown in **Figure 2**, given an input feature, a 1×1 convolution is first applied to expand the channel dimension, followed by a split into two parallel branches. The identity branch *A* preserves the original information to facilitate stable gradient propagation, while the semantic branch *B* is used for feature transformation. Specifically, the semantic branch consists of *m* cascaded Local-Global Modeling Blocks (LGM Blocks), denoted as *β_n_*, where the feature propagation is defined as shown in Equation 1:

(1) $$B_{n}=\beta_{n}\left(B_{n-1}\right),\;n\in 1,2,\ldots ,m.$$

To improve feature diversity and alleviate information loss, the outputs from these intermediate LGM Blocks are progressively aggregated and concatenated with the identity branch. By preventing information loss across cascaded LGM blocks, this progressive aggregation preserves local details within the identity branch that would otherwise vanish during global modeling. Subsequently, the final output of the DSMA module is obtained through a lightweight 1 × 1 convolution.

At the core of the semantic branch, each LGM block enables efficient long-range dependency modeling with linear computational complexity. Specifically, local features are first extracted using a combination of a 1 × 1 convolution and a 3×3 depth-wise convolution, and then flattened and transposed into a sequence, which is subsequently processed by layer normalization (LN) and the Mamba layer.

**Figure 3:**
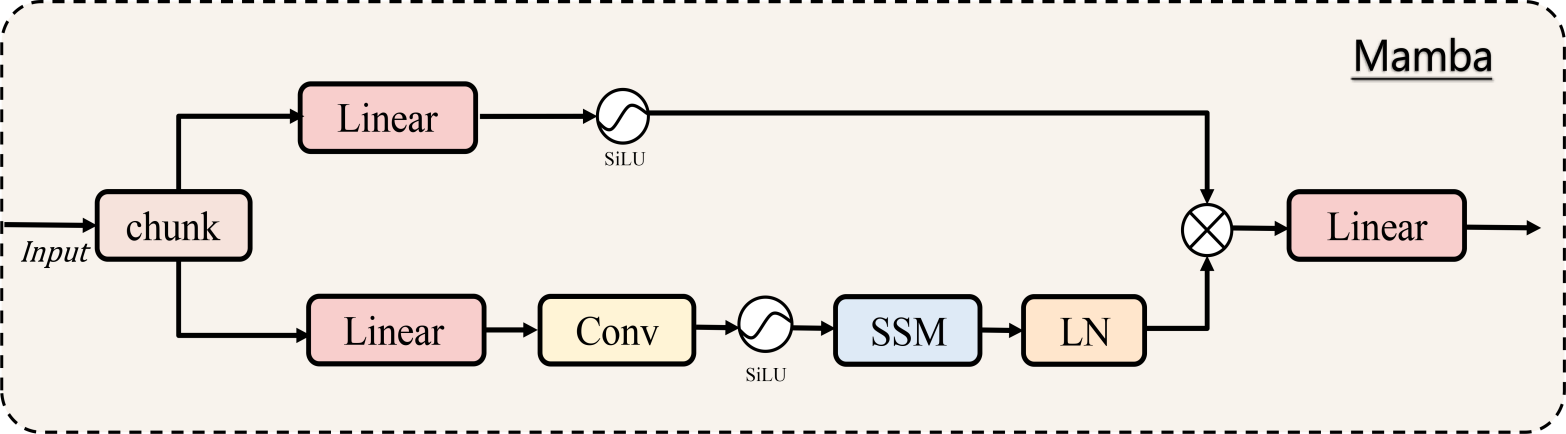
Overall architecture of the Mamba layer.

The architecture of the Mamba layer is shown in **Figure 3**. The input features are first split into two parallel branches along the feature dimension using the chunk function. In the primary branch, the features are processed via a linear projection and a convolution to capture local features, followed by a SiLU activation before being fed into the SSM. Leveraging a state transition mechanism, the SSM performs global modeling of sequential features. Its selective parameterization enables the network to dynamically focus on the key characteristics of lesions, thereby producing global semantic representations. In parallel, the gating branch generates a weight matrix via a linear transformation and a SiLU activation. By performing element-wise multiplication to fuse the outputs of these two branches, this gating mechanism adaptively reweights the feature space and enhances lesion-related representations.

In summary, DSMA improves contextual modeling while preserving local detail, which may help distinguish lesions from ambiguous background regions in breast ultrasound images.

### 2.5 Structure-aware axial attention

In ultrasound images, lesions often present anisotropic morphology and direction-dependent boundary appearances. Conversely, background speckle noise generally manifests as isotropic patterns. Conventional spatial attention mechanisms heavily rely on global pooling, which inherently dilutes these critical directional structural features. To preserve directional cues and maintain structural fidelity, we introduced the SAA module, which explicitly models spatial dependencies along orthogonal orientations.

**Figure 4:**
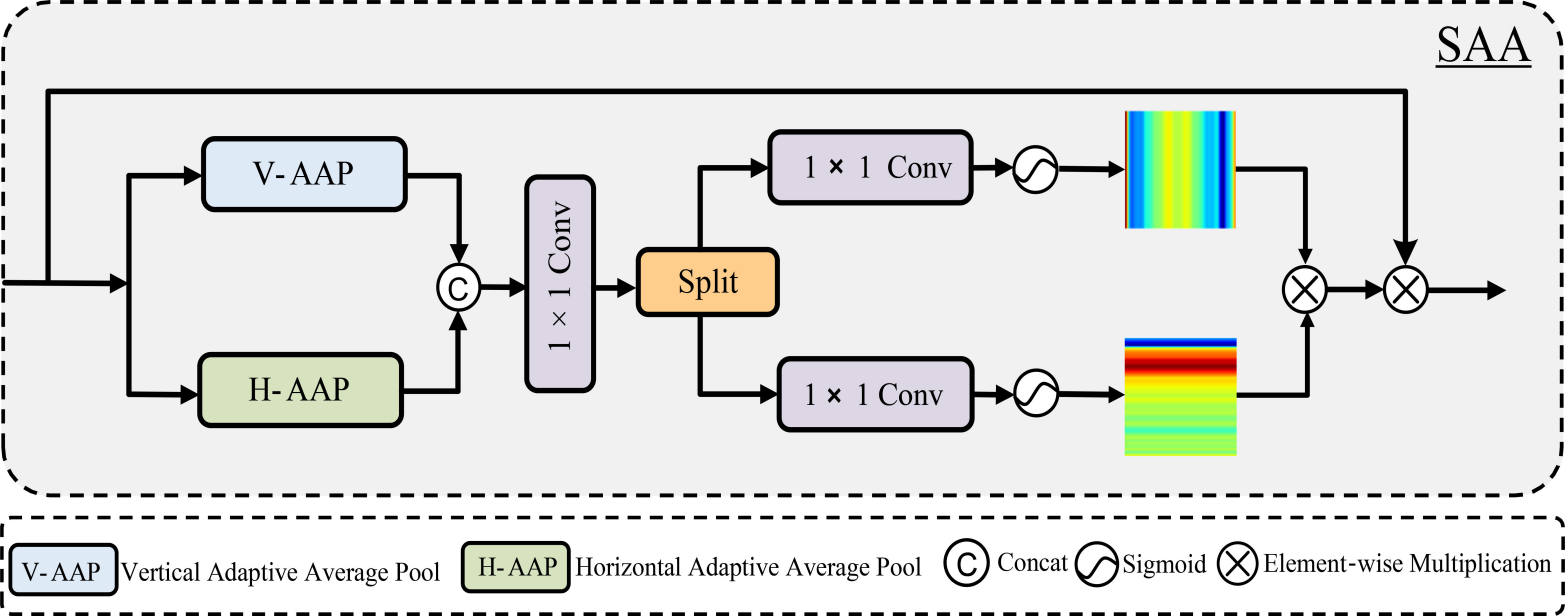
Overall architecture of the proposed Structure-aware Axial Attention module.

As illustrated in **Figure 4**, SAA captures features independently along the horizontal and vertical axes, thereby imposing directional constraints on feature aggregation. This decoupled directional aggregation strategy effectively highlights structures with strong line-like continuity while simultaneously suppressing isotropic random noise, enabling the network to better preserve boundary continuity and morphological consistency during feature refinement.

Formally, given an input feature map $I\in {\mathbb{R}}^{C\times H\times W}$, SAA employs adaptive average pooling along the vertical and horizontal axes to generate two distinct directional descriptors, denoted as $P_{H}\left(I\right)\in {\mathbb{R}}^{C\times H\times 1}$ and $P_{V}\left(I\right)\in {\mathbb{R}}^{C\times 1\times W}$, respectively. These descriptors are subsequently concatenated along the spatial dimension and transformed via a 1×1 convolution, followed by batch normalization (BN) and ReLU activation function to facilitate cross-channel feature interaction, as shown in Equation 2:

(2) $$F_{mid}=ReLU\left(BN\left(Conv_{1\times 1}\left(\left[P_{H}\left(I\right)∥P_{V}\left(I\right)\right]\right)\right)\right),$$

where [||] denotes the spatial concatenation operation, and *Conv*_1×1_ represents a 1×1 convolution layer. Subsequently, the intermediate feature *F_mid_* is split back into two directional components along the spatial axes. Each component is individually processed by a dedicated 1×1 convolution and a sigmoid function to generate the horizontal and vertical attention weights, respectively. The final output is obtained by element-wise multiplying the original input feature map *I* with these complementary directional weights.

Unlike traditional spatial aggregation methods that treat spatial dimensions indiscriminately, SAA integrates directional priors directly into the feature aggregation paradigm. This design is consistent with the anisotropic appearance of many breast lesions in ultrasound images. By decoupling spatial dependencies across orthogonal orientations, SAA can preserve directional structural cues and may improve boundary-related feature representation in degraded images.

## 3 Results

### 3.1 Quantitative comparison and efficiency analysis

To evaluate the effectiveness of the proposed method, we compare it with several representative detection approaches on the BUV and WH-BUS datasets, including YOLOv8 Varghese and M. (14), YOLOv10 Wang et al. (15), YOLOv11 Khanam and Hussain (16), YOLOv12 Tian et al. (12), YOLOv13 Lei et al. (13), FCM Xiao et al. (4), RT-DETR Zhao et al. (21), and Mamba-YOLO Wang et al. (27). All methods are trained and evaluated under the same experimental settings to ensure a fair comparison.

**Tables 1** and **2** present the quantitative comparison results on the BUV and WH-BUS datasets, respectively, while **Table 3** reports the computational complexity and inference efficiency of different methods. Overall, the proposed method provided a reasonable balance between detection accuracy and computational efficiency, suggesting its potential for real-time breast ultrasound analysis under resource-constrained settings.

**Table 1:** Quantitative comparison of different methods on the BUV dataset.

Methods	mAP	mAP50	Precision	Recall
YOLOv8	0.5389 ± 0.0034	0.9027 ± 0.0078	0.8691 ± 0.0368	0.8178 ± 0.0160
YOLOv10	0.5241 ± 0.0086	0.8809 ± 0.0114	0.8462 ± 0.0132	0.8070 ± 0.0216
YOLOv11	0.5377 ± 0.0077	0.9084 ± 0.0090	0.8582 ± 0.0103	0.8356 ± 0.0164
YOLOv12	0.5511 ± 0.0029	0.9096 ± 0.0028	0.8911 ± 0.0067	0.8185 ± 0.0076
YOLOv13	0.5511 ± 0.0063	0.9137 ± 0.0041	0.8822 ± 0.0213	0.8213 ± 0.0110
FCM	0.5181 ± 0.0013	0.8917 ± 0.0049	0.8767 ± 0.0184	0.8069 ± 0.0139
RT-DETR	0.5144 ± 0.0060	0.8711 ± 0.0051	0.8858 ± 0.0226	0.8163 ± 0.0208
Mamba-YOLO	0.5456 ± 0.0050	0.9074 ± 0.0113	0.8902 ± 0.0070	0.8248 ± 0.0182
Ours	0.5530 ± 0.0050	0.9086 ± 0.0066	0.8875 ± 0.0145	0.8136 ± 0.0186

**Table 2:** Quantitative comparison of different methods on the WH-BUS dataset.

Methods	mAP	mAP50	Precision	Recall
YOLOv8	0.7385 ± 0.0122	0.9338 ± 0.0072	0.9001 ± 0.0118	0.8637 ± 0.0158
YOLOv10	0.7354 ± 0.0077	0.9240 ± 0.0099	0.8877 ± 0.0112	0.8496 ± 0.0128
YOLOv11	0.7497 ± 0.0129	0.9425 ± 0.0046	0.9057 ± 0.0102	0.8829 ± 0.0079
YOLOv12	0.7680 ± 0.0054	0.9499 ± 0.0055	0.9214 ± 0.0053	0.8969 ± 0.0085
YOLOv13	0.7655 ± 0.0088	0.9526 ± 0.0058	0.9200 ± 0.0071	0.8984 ± 0.0098
FCM	0.7194 ± 0.0082	0.9247 ± 0.0086	0.8965 ± 0.0039	0.8600 ± 0.0097
RT-DETR	0.7064 ± 0.0200	0.9210 ± 0.0147	0.9122 ± 0.0132	0.8654 ± 0.0293
Mamba-YOLO	0.7301 ± 0.0096	0.9358 ± 0.0073	0.9043 ± 0.0054	0.8700 ± 0.0135
Ours	0.7675 ± 0.0059	0.9520 ± 0.0039	0.9208 ± 0.0080	0.8947 ± 0.0046

**Table 3:** Quantitative comparison of computational cost.

Methods	FPS	Params (M)	FLOPs (G)
YOLOv8	400.00	3.01	8.1
YOLOv10	312.50	2.69	8.2
YOLOv11	322.58	2.58	6.3
YOLOv12	243.90	2.51	5.8
YOLOv13	175.44	2.45	6.2
FCM	344.83	2.89	22.8
RT-DETR	78.13	31.99	103.4
Mamba-YOLO	135.14	5.98	13.6
Ours	161.29	2.50	6.4

On the BUV dataset, the proposed method achieved the highest mAP of 0.5530 among all compared approaches. Compared with YOLOv8, the mAP improved from 0.5389 to 0.5530 while maintaining comparable precision and recall. Compared with the baseline YOLOv13, although the improvement in mAP was relatively modest, the proposed method preserved balanced detection performance across repeated experiments. In addition, the proposed method achieved competitive localization accuracy under stricter IoU evaluation, suggesting that the joint modeling of contextual dependencies and structural information contributes positively to lesion localization in noisy ultrasound images. Notably, these results were achieved using only 2.50M parameters and 6.4 GFLOPs, indicating that the proposed method maintains lightweight deployment characteristics while improving feature representation capability.

On the WH-BUS dataset, the proposed method achieved an mAP of 0.7675 and an mAP50 of 0.9520, showing performance comparable to YOLOv12 and YOLOv13. Although YOLOv12 achieved a slightly higher mAP by a narrow margin, the proposed method exhibited smaller fluctuations in recall across different random seeds, indicating relatively stable optimization behavior. Compared with RT-DETR, the proposed method achieved a favorable trade-off between detection performance and computational cost and parameter complexity. In particular, RT-DETR required 31.99M parameters and 103.4 GFLOPs, whereas the proposed method reduced the model complexity to 2.50M parameters and 6.4 GFLOPs while maintaining competitive detection accuracy. Although several lightweight YOLO variants achieved faster inference speed, they mainly rely on conventional convolutional feature extraction and do not explicitly incorporate contextual aggregation or structural modeling mechanisms.

**Figure 5:**
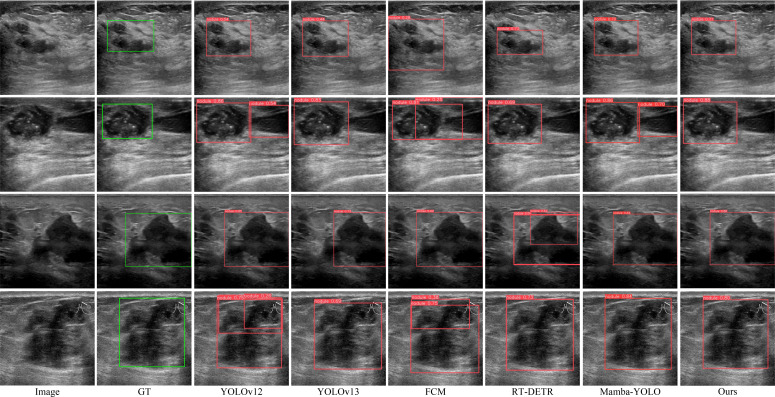
Qualitative comparison of breast lesion detection results.

**Figure 5** presents representative qualitative comparisons under several clinically challenging scenarios. As shown in the first row, lesions with blurred boundaries remain challenging for most detectors. FCM and RT-DETR occasionally produced bounding boxes with unstable scales or shifted localization results, whereas the proposed method generated comparatively tighter and more lesion-centered predictions. In the second row, several methods incorrectly responded to acoustic shadowing regions adjacent to the lesion, indicating limited discrimination between ultrasound artifacts and true anatomical structures. In contrast, the proposed method showed fewer false-positive responses in these examples. The third and fourth rows further demonstrate detection performance under heterogeneous echogenicity and irregular lesion morphology. Existing approaches occasionally generated incomplete localization or duplicated predictions, reflecting insufficient modeling of complex intra-lesion patterns. The proposed method maintained relatively consistent localization behavior across these challenging cases, producing bounding boxes that more closely matched the ground-truth annotations. These examples suggest that combining contextual aggregation with directional structural modeling may improve localization stability under complex ultrasound appearances.

### 3.2 Ablation study

#### 3.2.1 Effectiveness of DSMA and SAA

To evaluate the contribution of each proposed module, ablation experiments were conducted on the BUV and WH-BUS datasets using YOLOv13 as the baseline detector. DSMA and SAA were introduced individually and jointly into the baseline model. The quantitative results were reported in **Table 4** and **5**, where all metrics were presented as the mean ± standard deviation over five independent runs.

**Table 4:** Ablation results of the proposed modules on the BUV dataset.

Methods	mAP	mAP50	Precision	Recall
Baseline	0.5511 ± 0.0063	0.9137 ± 0.0041	0.8822 ± 0.0213	0.8213 ± 0.0110
+DSMA	0.5504 ± 0.0042	0.9047 ± 0.0078	0.8871 ± 0.0105	0.8131 ± 0.0085
+SAA	0.5519 ± 0.0078	0.9087 ± 0.0051	0.8802 ± 0.0143	0.8171 ± 0.0100
Ours	0.5530 ± 0.0050	0.9086 ± 0.0066	0.8875 ± 0.0145	0.8136 ± 0.0186

**Table 5:** Ablation results of the proposed modules on the WH-BUS dataset.

Methods	mAP	mAP50	Precision	Recall
Baseline	0.7655 ± 0.0088	0.9526 ± 0.0058	0.9200 ± 0.0071	0.8984 ± 0.0098
+DSMA	0.7660 ± 0.0039	0.9522 ± 0.0040	0.9176 ± 0.0064	0.9004 ± 0.0081
+SAA	0.7551 ± 0.0122	0.9477 ± 0.0065	0.9086 ± 0.0116	0.8898 ± 0.0127
Ours	0.7675 ± 0.0059	0.9520 ± 0.0039	0.9208 ± 0.0080	0.8947 ± 0.0046

On the BUV dataset, adding DSMA slightly increased precision from 0.8822 to 0.8871, while mAP and recall showed minor decreases. This result suggests that DSMA may help reduce some ambiguous responses, but its isolated effect on overall detection accuracy is limited under this dataset setting. Adding SAA alone produced a small mAP improvement from 0.5511 to 0.5519, indicating that directional structural modeling provides a modest benefit for lesion representation. When DSMA and SAA were combined, the model achieved the highest mAP and precision among the four settings, although the improvement over the baseline remained relatively small. These results indicate that the two modules provide complementary effects, but the gain on BUV should be interpreted conservatively.

On the WH-BUS dataset, DSMA alone slightly improved mAP and recall compared with the baseline, whereas SAA alone led to a decrease in all reported metrics. This suggests that the effectiveness of a single structural attention module may depend on the dataset distribution and lesion appearance characteristics. Nevertheless, the joint use of DSMA and SAA achieved the highest mAP of 0.7675 and the highest precision of 0.9208, while maintaining competitive recall. The standard deviations across repeated runs also indicate that the proposed configuration has relatively stable performance.

**Table 6** reports the computational cost of different module combinations. Compared with the baseline, the proposed model increased the number of parameters from 2.45M to 2.50M and FLOPs from 6.2G to 6.4G. The inference speed decreased from 175.44 FPS to 161.29 FPS, but remained within a real-time range. These results suggest that the proposed modules introduce only a small computational overhead while providing measurable improvements in feature representation and detection performance.

**Table 6:** Computational efficiency comparison of different module configurations.

Methods	FPS	Params (M)	FLOPs (G)
Baseline	175.44	2.45	6.2
+DSMA	163.93	2.45	6.3
+SAA	172.41	2.50	6.3
Ours	161.29	2.50	6.4

**Figure 6:**
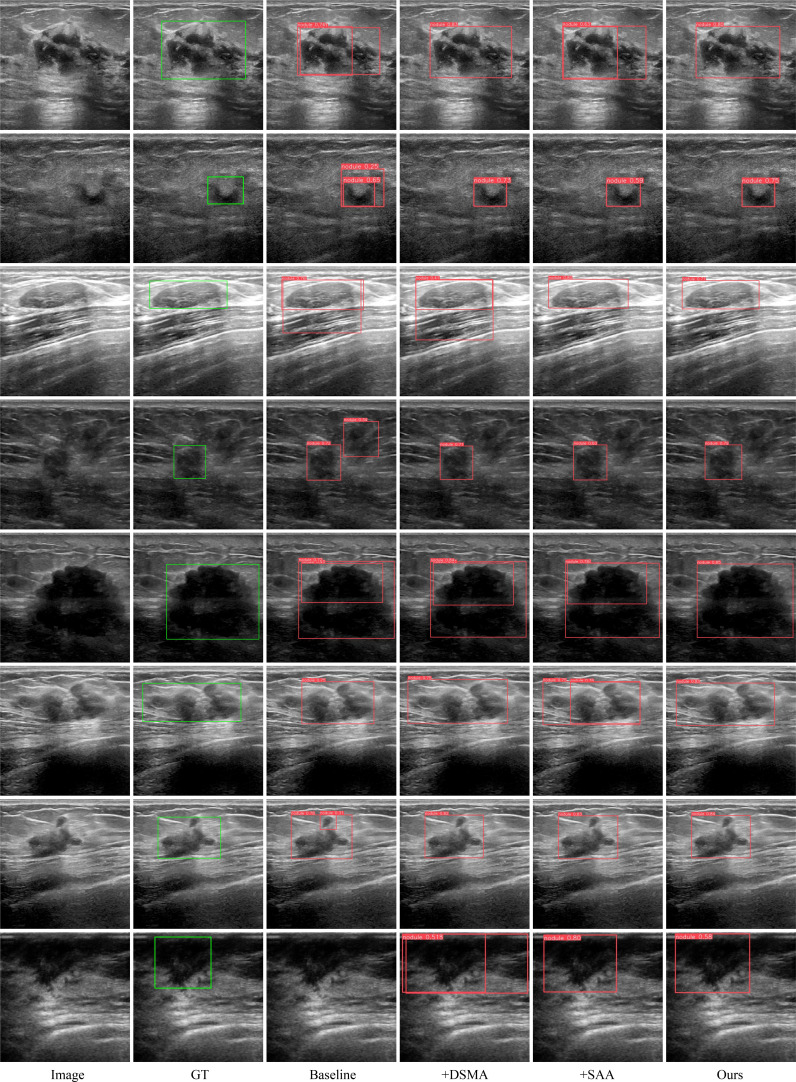
Qualitative ablation results of module combinations.

In addition, **Figure 6** shows representative qualitative ablation results under challenging ultrasound imaging conditions. In cases with low contrast, blurred boundaries, or heterogeneous internal echoes, the baseline detector occasionally produced shifted boxes, incomplete coverage, or additional false-positive responses. Introducing DSMA reduced some unstable responses in surrounding tissues, while SAA tended to produce tighter predictions around lesion-related structures in several examples. The full model generally yielded more consistent localization results and better agreement with the annotated lesion regions. These observations are consistent with the quantitative results and support the complementary roles of DSMA and SAA in contextual modeling and structural perception.

#### 3.2.2 Analysis of module deployment strategies

To investigate the influence of module placement, additional ablation experiments were conducted by inserting DSMA and SAA into different network stages. The results on the BUV and WH-BUS datasets are summarized in **Tables 7** and **8**. Here, “SB” and “DB” denote shallow and deep backbone stages, respectively, while “All” indicates deployment across both backbone and neck stages.

**Table 7:** Ablation study of different DSMA and SAA deployment strategies on the BUV dataset.

Setting	DSMA	SAA	Params (M)	FLOPs (G)	mAP	mAP50	Precision	Recall
Baseline	–	–	2.45	6.2	0.556	0.908	0.856	0.830
Proposed	SB	DB	2.50	6.4	0.562	0.925	0.912	0.806
SAA(B)	SB	SB+DB	2.52	6.5	0.550	0.909	0.892	0.805
SAA(All)	SB	All	2.82	7.0	0.557	0.904	0.819	0.844
DSMA(B)	SB+DB	DB	2.55	6.6	0.557	0.913	0.894	0.822
DSMA(All)	All	DB	2.72	6.5	0.554	0.908	0.854	0.834

**Table 8:** Ablation study of different DSMA and SAA deployment strategies on the WH-BUS dataset.

Setting	DSMA	SAA	Params (M)	FLOPs (G)	mAP	mAP50	Precision	Recall
Baseline	–	–	2.45	6.2	0.755	0.943	0.918	0.877
Proposed	SB	DB	2.50	6.4	0.777	0.956	0.922	0.902
SAA(B)	SB	SB+DB	2.52	6.5	0.756	0.941	0.910	0.882
SAA(All)	SB	All	2.82	7.0	0.751	0.952	0.918	0.893
DSMA(B)	SB+DB	DB	2.55	6.6	0.761	0.945	0.922	0.884
DSMA(All)	All	DB	2.72	6.5	0.759	0.951	0.918	0.903

In our proposed configuration, DSMA is placed in shallow backbone stages, while SAA is inserted into deeper backbone layers. This design is based on the different roles of the two modules. DSMA is intended to enhance spatial dependency modeling and local contextual aggregation, which is useful in early feature extraction stages where fine-grained textures and boundary-related details are still retained. SAA is designed to refine directional structural responses and is therefore more suitable for deeper layers, where lesion-related semantic features are more developed.

On the BUV dataset, our proposed deployment strategy achieved the highest mAP of 0.562, with 2.50M parameters and 6.4 GFLOPs. Compared with the baseline, it also improved mAP50 and precision, although recall was slightly lower. On the WH-BUS dataset, our proposed configuration also achieved the best mAP of 0.777 and improved both mAP50 and recall compared with the baseline. These results indicate that the asymmetric deployment of DSMA and SAA provides a reasonable balance between detection accuracy and computational cost.

However, expanding SAA to both shallow and deep backbone stages did not bring further improvement. Deploying SAA across all stages increased the computational cost to 7.0 GFLOPs and resulted in lower mAP on both datasets. A similar trend was observed for DSMA. Extending DSMA into deeper stages or all stages slightly improved recall in some settings, but the overall mAP did not exceed that of our proposed configuration. These findings suggest that simply increasing the number of attention modules does not necessarily improve detection performance. Excessive deployment may introduce redundant feature interactions or interfere with multi-scale feature fusion, especially for ultrasound images where subtle lesion boundaries and local texture variations are important.

#### 3.2.3 Sensitivity and replacement analysis

The sensitivity of the number of LGM Blocks in DSMA was further evaluated, as shown in **Table 9**. When *n* = 1, the model achieved the best trade-off between accuracy and efficiency, with 2.50M parameters and 6.4 GFLOPs. Increasing the number of LGM Blocks to 3 raised the computational cost to 6.9 GFLOPs but led to decreased mAP on both BUV and WH-BUS datasets. When *n* = 5, the mAP on the BUV dataset recovered slightly to 0.562, but precision remained lower than that of *n* = 1, and the computational cost further increased to 7.3 GFLOPs. These results indicate that stacking more LGM Blocks does not consistently improve performance and may introduce unnecessary redundancy. Therefore, *n* = 1 was selected in the final model.

**Table 9:** Sensitivity analysis of the number of LGM Blocks (n) on the BUV and WH-BUS datasets.

LGM block	Params (M)	FLOPs (G)	BUV				WH-BUS			
			mAP	mAP50	Precision	Recall	mAP	mAP50	Precision	Recall
n=1	2.50	6.4	0.562	0.925	0.912	0.806	0.777	0.956	0.922	0.902
n=3	2.53	6.9	0.543	0.911	0.868	0.835	0.758	0.950	0.919	0.895
n=5	2.54	7.3	0.562	0.92	0.868	0.820	0.765	0.950	0.919	0.895

**Table 10** compares the proposed modules with several lightweight alternative designs. Replacing SAA with CBAM Woo et al. (32) or ECA Wang et al. (33) led to lower mAP on both datasets, although CBAM achieved relatively high recall on the BUV dataset. Similarly, replacing DSMA with DSC3K2 reduced mAP compared with the proposed full DSMA+SAA configuration. On the BUV dataset, the proposed combination achieved the highest mAP, mAP50, and precision, while DSC3K2+SAA obtained higher recall. On the WH-BUS dataset, the proposed method achieved the highest mAP, whereas DSC3K2+SAA showed slightly higher mAP50, precision, and recall. These results suggest that the proposed DSMA and SAA combination provides a better overall accuracy-efficiency trade-off, although some alternative modules may be advantageous for specific metrics.

**Table 10:** Performance comparison with alternative lightweight modules.

DSMA	SAA	Params (M)	FLOPs (G)	BUV				WH-BUS			
				mAP	mAP50	Precision	Recall	mAP	mAP50	Precision	Recall
DSMA	CBAM	2.5	6.4	0.556	0.916	0.865	0.838	0.773	0.954	0.916	0.896
DSMA	ECA	2.45	6.3	0.533	0.899	0.899	0.812	0.756	0.946	0.917	0.886
DSC3K2	SAA	2.5	6.3	0.548	0.907	0.867	0.852	0.766	0.959	0.925	0.904
DSMA	SAA	2.5	6.4	0.562	0.925	0.912	0.806	0.777	0.956	0.922	0.902

Therefore, the ablation results indicate that DSMA and SAA are most effective when used in a complementary and stage-specific manner. DSMA contributes to early contextual feature aggregation, while SAA refines deeper structural representations. The proposed configuration improves detection performance with limited additional computational cost, and the qualitative results further support its benefit for lesion localization under complex ultrasound appearances.

### 3.3 Interpretability and feature response analysis

**Figure 7:**
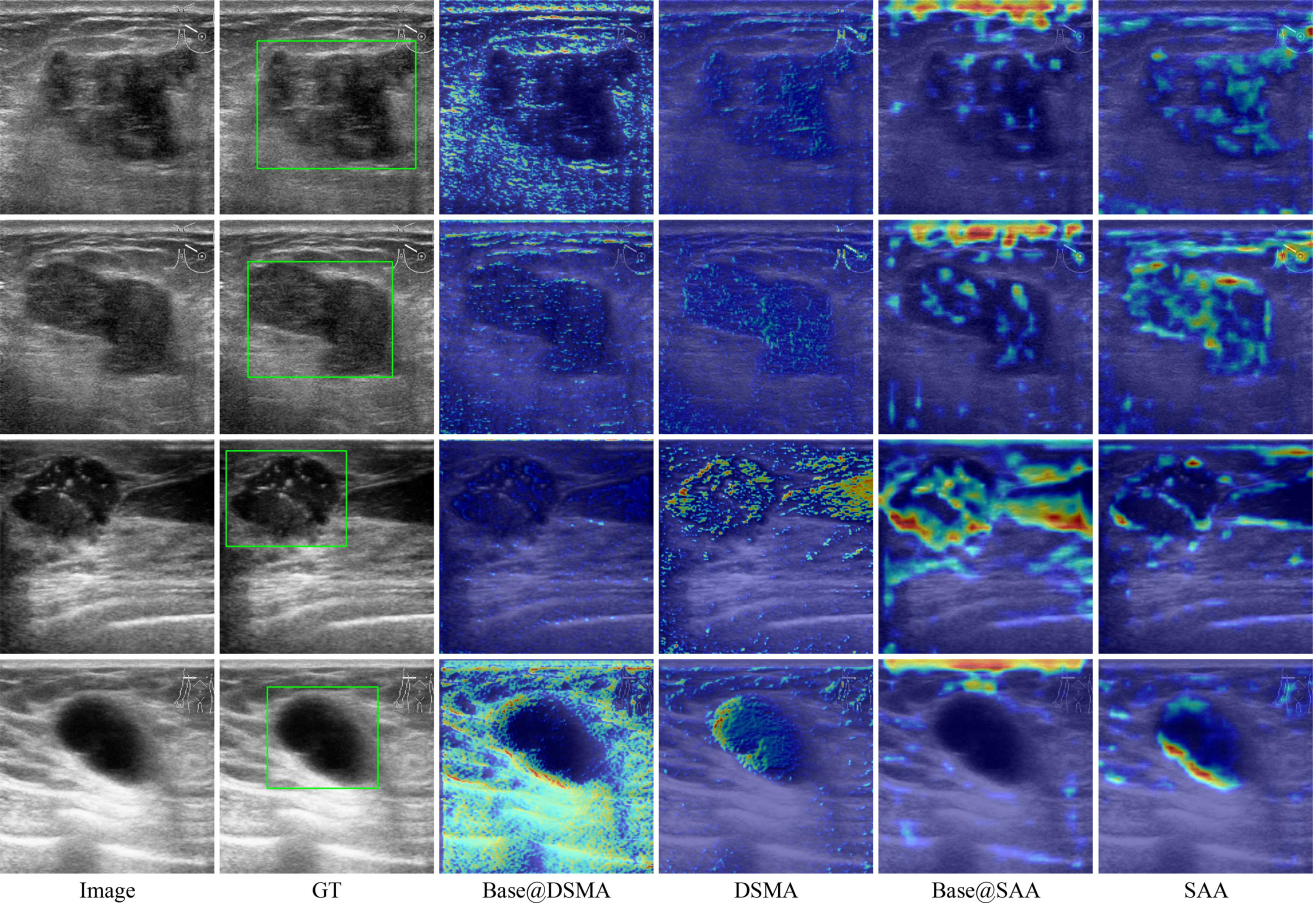
Feature response visualization of DSMA and SAA modules.

To examine the representation behavior of the proposed modules, feature response visualizations of DSMA and SAA are shown in **Figure 7**, where “Base@DSMA” and “Base@SAA” denote the baseline feature responses at the corresponding module insertion positions. From left to right, the columns show the original image, ground-truth annotation, baseline feature response before inserting DSMA, feature response after introducing DSMA, baseline feature response before introducing SAA, and feature response after introducing SAA. As illustrated, compared with the corresponding baseline, the responses produced by DSMA are generally less scattered and show higher activation around lesion-related regions and their surrounding context. Meanwhile, activations from irrelevant background textures are reduced in several cases, suggesting that DSMA contributes to contextual feature aggregation and helps improve the consistency of lesion-related responses under heterogeneous ultrasound appearances. In contrast, for SAA, the responses tend to be more structurally organized, with relatively clearer activation around lesion margins and adjacent tissue interfaces. This pattern is consistent with the motivation of introducing structure-aware attention to model directional dependencies in breast ultrasound images. Overall, these qualitative results indicate that DSMA mainly enhances contextual representation, whereas SAA provides complementary structural sensitivity.

**Figure 8:**
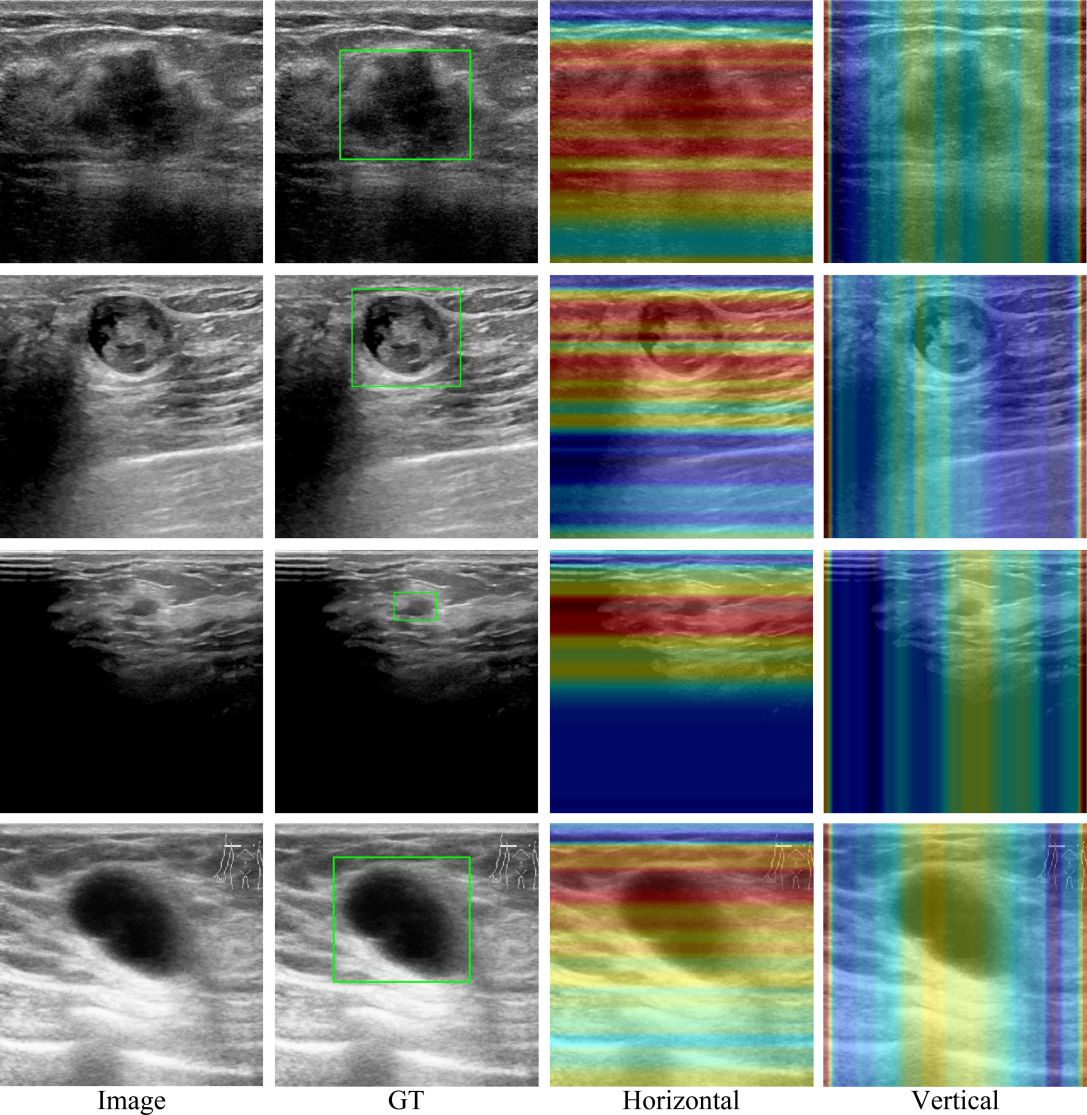
Visualization of the directional attention responses generated by the SAA module.

To further analyze the internal behavior of SAA, **Figure 8** presents the attention maps generated by the horizontal and vertical branches. The horizontal branch produces band-like responses along the lateral direction and highlights tissue structures crossing the lesion and its neighboring regions. This indicates that the horizontal branch is sensitive to lateral continuity and boundary-related texture transitions. In contrast, the vertical branch shows elongated responses along the depth direction, with activations passing through the lesion region and extending to deeper acoustic areas in several examples. Such behavior is consistent with the depth-dependent characteristics of ultrasound imaging, where posterior attenuation, shadowing, or enhancement may appear along the beam direction. These observations suggest that SAA can separately model horizontal and vertical structural dependencies. The two directional branches provide complementary information, the horizontal branch emphasizes lateral tissue continuity, while the vertical branch captures depth-related structural responses. This anisotropic modeling is relevant for breast ultrasound analysis because lesion morphology, tissue interfaces, and acoustic artifacts often exhibit direction-dependent patterns.

**Figure 9:**
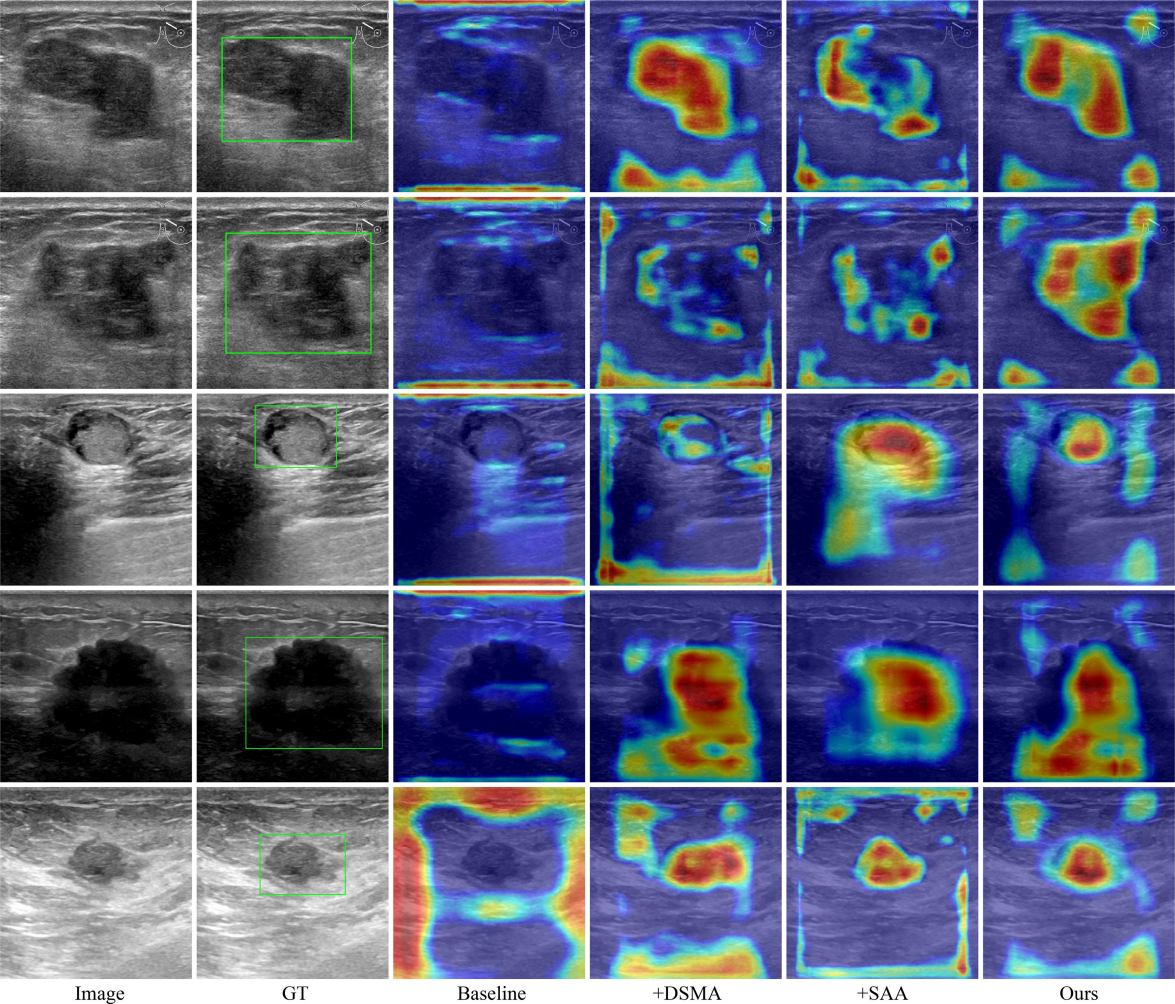
Detection head response visualization under different module configurations.

In addition, **Figure 9** visualizes the detection head responses under different module configurations to evaluate their influence on lesion localization. The baseline model produces relatively dispersed activations in several cases, and some responses extend beyond the lesion region or appear in surrounding background tissues, especially in low-contrast or heterogeneous areas. After introducing DSMA, the high-response regions become more closely associated with the lesion area, although residual activations can still be observed in adjacent tissues. This suggests that DSMA improves context-dependent feature discrimination but does not fully resolve structural ambiguity. With SAA, the response maps show improved boundary awareness and more compact activation around lesion-related structures, which indicates that directional structural modeling helps refine localization cues. When DSMA and SAA are combined, the detection responses are generally more concentrated on the lesion region, with reduced background activation compared with the baseline. The activated areas also show better agreement with the annotated lesion regions in most examples. These visualization results suggest that the proposed modules enhance feature representation and lesion localization in a complementary manner.

### 3.4 Cross-dataset generalization

To evaluate the cross-dataset generalization capability of the proposed method, experiments were conducted between the BUV and WH-BUS datasets. In these experiments, the model trained on one dataset was directly evaluated on the other dataset without domain-specific fine-tuning. The results are reported in **Table 11**.

**Table 11:** Cross-dataset generalization results on the BUV and WH-BUS datasets.

Train	Test	mAP	mAP50	Precision	Recall
BUV	BUV	0.562	0.925	0.912	0.806
WH-BUS	BUV	0.549	0.916	0.895	0.857
WH-BUS	WH-BUS	0.817	0.967	0.942	0.947
BUV	WH-BUS	0.799	0.967	0.939	0.911

Compared with the in-domain setting on BUV, where the model achieved an mAP of 0.562, the model trained on WH-BUS and tested on BUV obtained an mAP of 0.549, corresponding to a relative decrease of 2.31%. Similarly, when transferring from BUV to WH-BUS, the mAP decreased from 0.817 in the in-domain WHBUS setting to 0.799, with a relative decrease of 2.20%. These results indicate that the proposed framework maintains relatively stable detection performance under cross-dataset evaluation. The performance differences are likely related to variations in imaging protocols, lesion appearance, annotation characteristics, and background tissue distributions between the two datasets. In particular, the lower overall mAP on BUV suggests that this dataset may contain more challenging cases, such as low-contrast lesions, heterogeneous parenchymal textures, and acoustic interference.

It is also worth noting that the recall on BUV increases from 0.806 in the in-domain setting to 0.857 under WH-BUS-to-BUV transfer, although precision and mAP decrease slightly. This suggests that the transferred model tends to detect more candidate lesion regions, but with a modest reduction in localization or classification precision. On WH-BUS, the cross-dataset model trained on BUV achieves the same mAP50 as the in-domain model, while showing a small decrease in mAP and recall. These findings suggest that the proposed framework preserves a certain degree of lesion localization ability across datasets, but its performance is still influenced by dataset-specific image characteristics.

In summary, the cross-dataset results show that the proposed method does not rely solely on dataset-specific texture patterns. The combination of DSMA and SAA may help the network learn more transferable contextual and structural representations, which contributes to relatively stable performance under heterogeneous ultrasound imaging conditions.

### 3.5 Noise robustness analysis

Breast ultrasound images are often degraded by speckle artifacts, acoustic scattering, defocus, motion-induced blur, and variations in acquisition quality, all of which can reduce lesion contrast and obscure boundary details. To evaluate the robustness of the proposed method under such challenging imaging conditions, we further conducted corruption-based experiments by introducing Gaussian noise, speckle noise, and Gaussian blur with different severity levels into the test images.

Following a commonly adopted robustness evaluation protocol, all models were trained exclusively on the original clean training set and directly tested on the corrupted images without any additional fine-tuning. Specifically, Gaussian noise was introduced to simulate random intensity perturbations, speckle noise was used to mimic the multiplicative interference inherently present in ultrasound imaging, and Gaussian blur was applied to emulate image quality degradation caused by defocus or motion artifacts. Through these experiments, we aimed to comprehensively assess the stability and generalization capability of the proposed model under realistic image degradation scenarios.

As shown in **Table 12**, the proposed method maintained stable performance across all tested corruption settings. Compared with the clean setting, the largest mAP decrease of the proposed method is 1.47%, observed under Gaussian blur with a kernel size of 7. Under severe speckle noise with variance 0.1, the proposed method achieved an mAP of 0.5567, corresponding to a 1.01% decrease from the clean setting. Under the same condition, it outperformed YOLOv13 and RT-DETR by 1.66% and 6.49% in mAP, respectively. Under strong Gaussian blur with kernel size 7, the proposed method obtained an mAP of 0.5541, showing only a limited decrease compared with the clean baseline.

**Table 12:** Quantitative robustness analysis under different ultrasound-related corruption conditions.

Noise type	Level	YOLOv13		RT-DETR		Mamba-YOLO		Ours	
		mAP	Drop	mAP	Drop	mAP	Drop	mAP	Drop
clean	–	0.5565	–	0.4941	–	0.5299	–	0.5624	–
Gaussian noise	0.01	0.5561	↓0.06%	0.4942	↑0.03%	0.5292	↓0.13%	0.5631	↑0.13%
Gaussian noise	0.03	0.5517	↓0.86%	0.4971	↑0.62%	0.5188	↓2.09%	0.5623	↓0.02%
Gaussian noise	0.05	0.5369	↓3.51%	0.5001	↑1.22%	0.4993	↓5.77%	0.5556	↓1.22%
Speckle noise	0.02	0.5559	↓0.09%	0.4940	↓0.01%	0.5292	↓0.13%	0.5623	↓0.02%
Speckle noise	0.05	0.5529	↓0.64%	0.4926	↓0.30%	0.5227	↓1.36%	0.5617	↓0.12%
Speckle noise	0.1	0.5401	↓2.94%	0.4918	↓0.47%	0.5032	↓5.04%	0.5567	↓1.01%
Gaussian blur	3	0.5596	↑0.56%	0.4941	↑0.01%	0.5393	↑1.77%	0.5638	↑0.24%
Gaussian blur	5	0.5569	↑0.07%	0.4956	↑0.31%	0.5421	↑2.30%	0.5619	↓0.10%
Gaussian blur	7	0.5524	↓0.73%	0.4954	↑0.28%	0.5316	↑0.32%	0.5541	↓1.47%

Compared with YOLOv13 and Mamba-YOLO, the proposed method shows a slower performance degradation trend under increasing Gaussian and speckle noise. For example, under Gaussian noise with *σ* = 0.05, YOLOv13 decreases by 3.51%, and Mamba-YOLO decreases by 5.77%, whereas the proposed method decreases by 1.22%. A similar trend can be observed under speckle noise, where the proposed method shows smaller mAP reductions at all tested noise levels. For Gaussian blur, all models exhibit relatively small performance variations, and slight improvements are observed in some cases. This may be because moderate smoothing suppresses high-frequency background fluctuations and speckle-like textures, although excessive blur can still weaken lesion boundary information.

**Figure 10:**
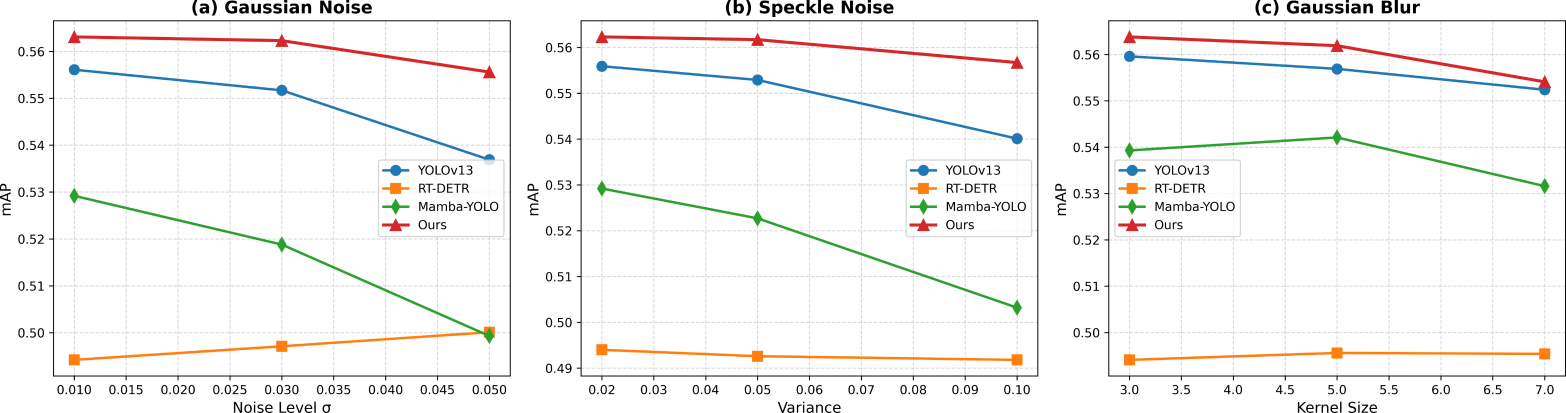
Robustness comparison under different corruptions.

**Figure 10** further illustrates the robustness trends under progressively increasing corruption intensity. RT-DETR shows relatively stable curves but maintains lower overall mAP across the tested settings. YOLOv13 achieves competitive performance under mild corruption but exhibits a clearer decrease under stronger Gaussian and speckle noise. Mamba-YOLO is more sensitive to Gaussian and speckle perturbations, while showing relatively favorable behavior under mild blur. In comparison, the proposed method achieves the highest or near-highest mAP under most corruption settings and maintains a relatively stable degradation trend.

The observed robustness may be attributed to the complementary roles of DSMA and SAA. DSMA enhances contextual aggregation over lesion-related regions, which may reduce the influence of isolated noisy responses. SAA introduces directional structural modeling, which can help preserve boundary-related and depth-related cues when image quality is degraded. Nevertheless, these experiments are based on synthetic corruptions and should be interpreted as controlled robustness analysis rather than a complete substitute for evaluation on prospectively collected low-quality clinical ultrasound images.

## 4 Discussion

In this study, we proposed a lightweight context-structure synergistic framework for breast ultrasound lesion detection. Experiments on the BUV and WH-BUS datasets showed that the proposed method achieved competitive detection performance while maintaining a compact parameter count and real-time inference speed. These results suggest that integrating contextual dependency modeling with structure-aware feature refinement offers a promising approach for breast ultrasound images, which are frequently challenged by speckle noise, blurred boundaries, acoustic artifacts, and heterogeneous tissue backgrounds.

This performance gain stems from the complementary attributes of the DSMA and SAA modules. Specifically, the DSMA module enables efficient long-range contextual aggregation, helping the network distinguish lesion-related features from background interference. Given that speckle artifacts and heterogeneous background tissues frequently mimic lesion morphologies in breast ultrasound, relying solely on local texture cues often leads to false positive interpretations. DSMA may alleviate this limitation by establishing global semantics across long-range spatial dependencies. By capturing this broad spatial envelope, DSMA may reduce discrete artifact-related responses that lack spatial coherence, while reinforcing the semantic consistency of true lesion structures. Complementarily, the SAA module explicitly captures directional structural information along the horizontal and vertical axes, which is highly relevant to BUS imaging characterized by the anisotropic distributions of lesion boundaries, tissue interfaces, and posterior acoustic patterns. The integration of these modules enables the detector to concurrently leverage semantic context and structural continuity.

Ablation studies and qualitative visualizations further support this complementary behavior. Quantitative analysis showed that using either module alone led to only marginal changes, suggesting that contextual aggregation and directional structural modeling provide complementary effects. The deployment ablation also indicated that placing DSMA in the shallow stages and SAA in deeper layers offered a favorable trade-off between detection accuracy and computational overhead. Feature response maps further illustrate that DSMA effectively diminishes diffuse activations within background tissues, whereas SAA generates highly organized, directional responses along lesion boundaries and depth-dependent structures. Consequently, the final detection head produces more compact, lesion-centered predictions than the baseline.

The framework also showed relatively stable performance in robustness and cross-dataset analysis. In cross-dataset experiments without domain-specific fine-tuning, the model maintained stable mAP metrics across both transfer directions, indicating that its feature representation is less dependent on dataset-specific textural patterns. Moreover, under synthetic perturbations including Gaussian noise, speckle noise, and Gaussian blur, the proposed framework exhibited smaller performance degradation than baseline detectors, which is consistent with the intended roles of DSMA in contextual aggregation and SAA in structural feature preservation. Nevertheless, the remaining cross-dataset performance gap highlights the persistent challenges posed by variations in imaging protocols and lesion distributions, while the synthetic noise analysis serves as a controlled evaluation rather than a complete surrogate for degraded, real-world clinical scans.

**Figure 11:**
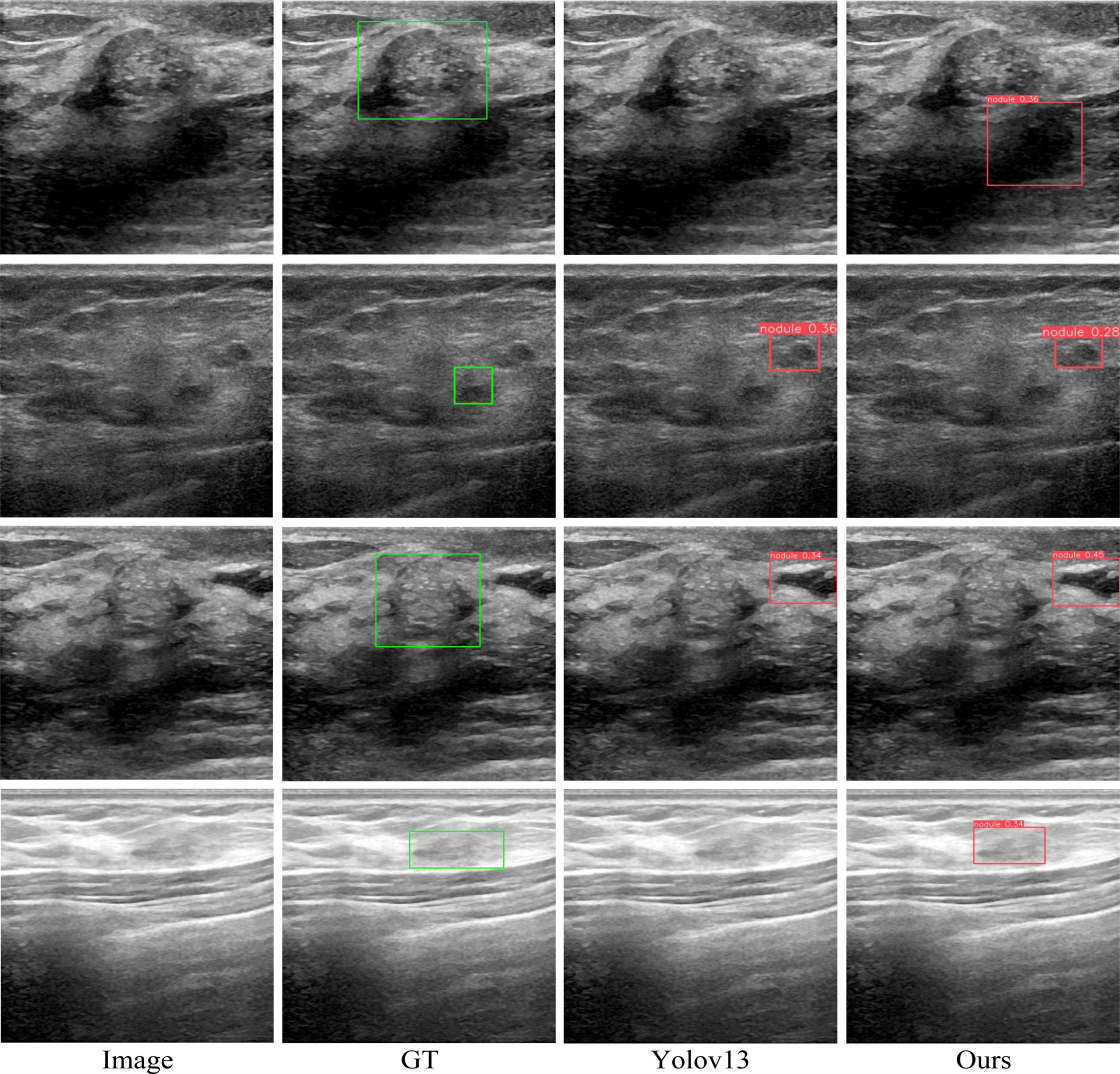
Representative failure cases on challenging breast ultrasound images.

Despite these results, several limitations should be acknowledged. First, the current framework processes each image independently, without exploiting the temporal continuity inherent in clinical ultrasound scanning. Second, the evaluation was conducted on two public datasets with limited diversity in scanners, operators, acquisition protocols, and lesion subtypes. Third, failure cases still occur in highly challenging scenarios. As shown in **Figure 11**, the model occasionally misses very small or low-contrast lesions, particularly under severe speckle noise, acoustic shadowing, or indistinct boundaries. In some cases, both the baseline and the proposed method produce inaccurate or missed detections, highlighting the persistent difficulty of robust lesion detection in extremely ambiguous ultrasound images.

Future work will focus on addressing the current limitations. First, we plan to extend the framework to video-based detection by incorporating temporal feature aggregation, which can exploit inter-frame consistency and anatomical continuity during dynamic ultrasound scanning. Second, uncertainty estimation will be integrated to identify low-confidence predictions, potentially providing more informative outputs to assist radiologists in clinical decision-making. Third, larger-scale multi-center validation and prospective clinical evaluations are essential to assess the model’s generalizability across diverse scanners, operators, and patient populations. In addition, although the proposed lightweight context-structure synergistic framework demonstrates competitive performance for breast ultrasound lesion detection, further validation is required before it can be considered for routine clinical use.

## Data Availability

The original contributions presented in the study are included in the article/supplementary material. Further inquiries can be directed to the corresponding author.
